# Implementing My Abilities First for Children with Developmental Delays in Taiwan: A Strengths-Based, ICF-Informed Practice Report

**DOI:** 10.3390/children13030381

**Published:** 2026-03-09

**Authors:** Hua-Fang Liao, Yi-Ling Pan, Pei-Jung Wang, Yen-Tzu Wu, Ya-Tzu Liao, Verónica Schiariti

**Affiliations:** 1 School and Graduate Institute of Physical Therapy, College of Medicine, National Taiwan University, Taipei 100, Taiwan; hfliao@ntu.edu.tw (H.-F.L.); yenwu@ntu.edu.tw (Y.-T.W.); 2 Taiwan Society of ICF, Taipei 100, Taiwan; cynliao@gmail.com; 3 Division of Physical Therapy, Department of Physical Medicine and Rehabilitation, National Taiwan University Hospital, Taipei 100, Taiwan; r97428003@ntu.edu.tw; 4 Department of Physical Therapy, Asia University, Taichung 413, Taiwan; pjwang@asia.edu.tw; 5 Physical Therapy Center, National Taiwan University Hospital, Taipei 100, Taiwan; 6 School of Medical Sciences, University of Victoria, Victoria, BC V8P 5C2, Canada

**Keywords:** early childhood intervention, early childhood education, participation, children with developmental disabilities, paradigm shift, self-expression, rights

## Abstract

**Highlights:**

**What are the main findings?**

- My Abilities First (MAF) was successfully implemented at scale in Taiwan, with high training satisfaction, improved practitioner subjective competence, and increasing use of My Abilities Identification Cards (ABIDs).
- The ABID framework systematically integrates child-led strengths and voices, fostering greater self-expression and creating meaningful participation opportunities for children with developmental delays.

**What are the implications of the main findings?**

- MAF is a feasible and scalable, strengths-based, rights-oriented approach for integration into existing service systems for children with developmental delays.
- Integration of ABIDs into early intervention and education may facilitate sustained practice change toward participatory, family-centered, rights-based care.

**Abstract:**

This practice-based implementation report describes the adoption of the My Abilities First (MAF) initiative for children with developmental delays in Taiwan. Grounded in the International Classification of Functioning, Disability and Health (ICF) framework, MAF emphasizes a strengths-based, participatory, and human rights-oriented approach to early childhood intervention. The purpose of this report is to describe the development of the MAF framework and the details of its innovative, culturally sensitive implementation in Taiwan, using implementation science principles to support the national adoption of My Abilities ID Cards (ABIDs). Central to the MAF initiative is the ABID, a tool that empowers children to express their abilities, preferences, and support needs using their own voice or preferred mode of communication. Guided by implementation science, the MAF team in Taiwan engaged stakeholders in urban and rural centers, developed training programs, and integrated ABID into early intervention and special education systems. Preliminary outcomes indicate that from 2021 to 2025, 140 training sessions reached a total attendance of 6961. Notably, satisfaction with training was high (>95%), and practitioner subjective competence adopting positive language improved. The number of children under age 12 creating ABIDs grew to approximately 700. Preliminary evidence suggests that ABIDs might increase systematic adoption of children’s opinions in assessments and interventions. Qualitative feedback from parents and professionals highlights the contribution of ABIDs, ensuring self-expression, motivation, and meaningful participation. The pioneering Taiwanese experience demonstrates the feasibility and impact of MAF and ABIDs in promoting children’s rights and participation, offering practical insights for global adaptation in diverse contexts.

## 1. Introduction

Grounded in the International Classification of Functioning, Disability and Health (ICF) framework, activities and participation are widely recognized as the most meaningful health outcomes for children, youths, and families [1,2]. Within the ICF framework, participation refers to an individual’s involvement in life situations, encompassing attendance, engagement, and active contribution in meaningful activities across social and environmental contexts. Using the ICF, individuals with lived experience of disability, of all ages, consistently report what they are able to do in their everyday lives, within the settings where they live, learn, play, and work [1,2].

Adopting a lifespan approach that promotes positive childhood experiences—such as the use of positive language and supportive relationships—can lead to better participation and health outcomes in adulthood [3]. Using positive language means the intentional use of affirming, respectful, and capability-oriented terminology that emphasizes strengths and functional potential, rather than limitations. Accordingly, clinicians, leaders, and policymakers are increasingly encouraged to embrace strengths-based resilience-focused approaches, recognizing that all children experiencing a health condition and their families possess strengths to contribute meaningfully to society [4].

Knowing the benefits of using positive language in neuroscience and education [5,6,7,8], recent efforts have sought to create tools that adopt positive language in service settings. **“My Abilities First” (MAF)** was created with several objectives: (1) to shift attitudes towards disability by emphasizing abilities rather than deficits; (2) to facilitate the human right of self-expression for healthcare users; and (3) to promote a participatory approach in goal setting [9,10]. Of note, the human right of self-expression refers to the fundamental right of individuals to freely communicate their thoughts, opinions, preferences, and identities through verbal, nonverbal, written, artistic, or other forms of expression without discrimination or undue restriction, consistent with human rights principles and legal protections.

MAF invites healthcare users to create a **My Abilities ID Card (ABID)**. Through the ABID, individuals describe who they are, their abilities, preferences, and support needs using their preferred way of communication [9,10]. This tool aligns with the Convention on the Rights of the Child (CRC) and the Convention on the Rights of Persons with Disabilities (CRPD), as well as the Sustainable Development Goals (SDGs) for inclusive education [11]. Subsequently, ABIDs can be incorporated into healthcare, education, and social development records, contributing positive language to current data collection systems.

In this practice-based implementation report we sought to summarize the feasibility, reach and preliminary implementation progress of ABIDs in Taiwan. Therefore, the specific purposes of this article are threefold: (1) to describe the development of the MAF framework; (2) to illustrate how implementation science has been applied in Taiwan to support the adoption of the ABID; and (3) to provide recommendations for the global use of MAF.

## 2. My Abilities First—Global Context

The development of MAF was inspired by qualitative research with Canadian children with cerebral palsy. These children, regardless of impairment severity, consistently talked about their *abilities* and interests, contrasting with the deficit-oriented perspectives in healthcare contexts, often held by professionals and at times by parents/caregivers [10]. Consequently, MAF was created to honor children’s voices and opinions.

The CRC and the CRPD emphasize autonomy, independence, and dignity for all individuals, regardless of age or ability. However, in healthcare and education, the perspectives of children with disabilities or developmental delays are often overlooked. MAF and its cultural adaptations address this gap by systematically seeking and valuing the voices of children and youth.

MAF challenges traditional deficit-based views of disability by promoting an abilities- and rights-based approach in medical education and practice. It includes educational web-based animations and proposes the creation of the ABID, which can be integrated into health records. MAF has facilitated global collaboration among clinical and research teams, enabling shared learning and fostering positive attitudes toward disability.

Since its first publication in 2020 [12], MAF has been adopted in multiple international projects and campaigns to promote equity, diversity, and inclusion [10]. These initiatives invite individuals with disabilities to introduce themselves using their native language and preferred communication mode, resulting in a wealth of positive-language materials shared worldwide. As dissemination continues, more groups are expressing interest in adopting this approach, and individuals with disabilities are increasingly advocating for their abilities to be systematically recognized before focusing on their functional limitations.

Globally, MAF is a growing movement. Colleagues in Brazil, for example, have embraced this human rights-based approach, identifying MAF as a driving factor in promoting equity and participation, though noting that attitudinal barriers remain. Taiwan has pioneered the systematic integration of this tool within its early childhood intervention (ECI) and special education systems for children with developmental delays (DDs).

## 3. Development and Implementation of MAF in Taiwan

### 3.1. Context: ECI and ICF in Taiwan

Taiwan’s ECI system for preschoolers with DDs began in the 1980s [13]. Historically, services were fragmented and deficit-focused. However, with the promulgation of the People with Disabilities Rights Protection Act (2007), Child and Youth Welfare and Protection Act (2012), CRC and CRPD Implementation Act (2014), and Special Education Act (2019), and the adoption of the ICF for disability assessment, have promoted inclusive education and the adoption of the ICF framework for assessment and service provision [14]. Besides, awareness of societal participation and human rights has grown [15]. Taiwan has promoted MAF since 2020 to ensure children’s strengths are acknowledged first, moving away from a negative approach that only identifies disabilities [9]. Then, Taiwan introduced the ABID as a tool to empower children to articulate their strengths [9] for promoting participation and exercising the self-expression and self-determination rights included in the CRC [16].

### 3.2. The Tool: My Abilities ID Card (ABID)

**Figure 1:**
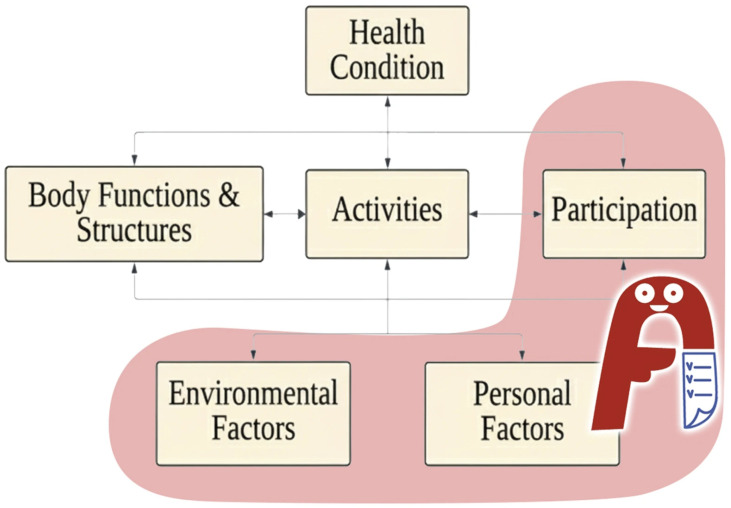
My Abilities First enhances social participation and serves as a facilitator of environmental and personal factors in the ICF framework.

By using positive language, the MAF and ABID serve as facilitators of environmental and personal factors promoting the identification of abilities related to participation. Figure 1 illustrates the ICF components highlighted by MAF. In addition, the MAF logo—
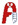
—of the project combines positive concepts including “F” (Facilitators for participation) and “A” (Abilities or strengths). A central heart symbolizes empathy, while eyes and a mouth invite people to “see and advocate” for strengths, exercising human rights.

**Figure 2:**
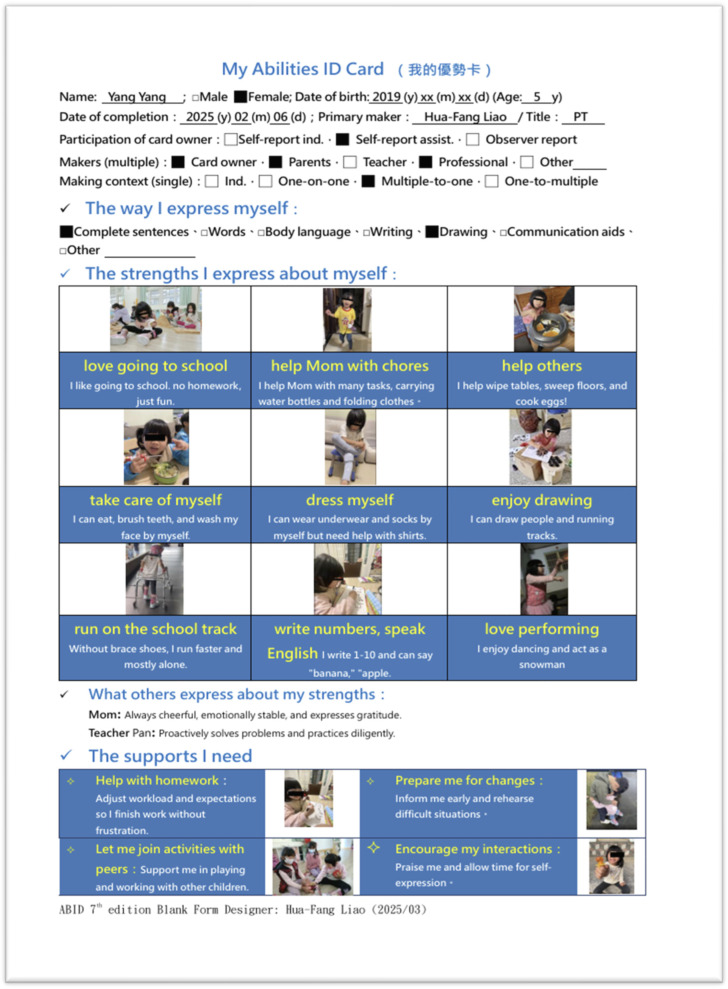
An example of a My Abilities ID Card of a 5-year-old girl with cerebral palsy.

Using a participatory approach, the current Taiwanese version of the ABID comprises four components (see Figure 2 for an example of “Yangyang (pseudo name)”, a 5-year-old girl with cerebral palsy):

1. StrengthsI express about myself: (e.g., “I can draw,” “I like performing”).
2. The supports I need: (e.g., “I need advance notice for schedule changes”).
3. The way I express myself.
4. What others express about my strengths.

To develop a visually engaging format of the ABID template forms to fit users’ needs in various settings and training contexts, the template forms underwent continuous refinement. From the trial version in 2021, and after incorporating feedback from families and professionals, the team revised the ABID format progressively. Currently, the Taiwanese ABID template 7.0 version consists of digital and paper forms, as well as an ABID online creation platform. The visually engaging format enables others to quickly understand the child’s abilities and support needs, shifting focus from limitations to strengths.

There are three levels of participation in creating the card: (1) self-report independently; (2) self-report with assistance (encouraged for most children); and (3) observer report (for those with severe communication disabilities). Participation in ABID development is encouraged as both an intervention outcome and a means to promote engagement [17].

### 3.3. Implementation Strategy

**Figure 3:**
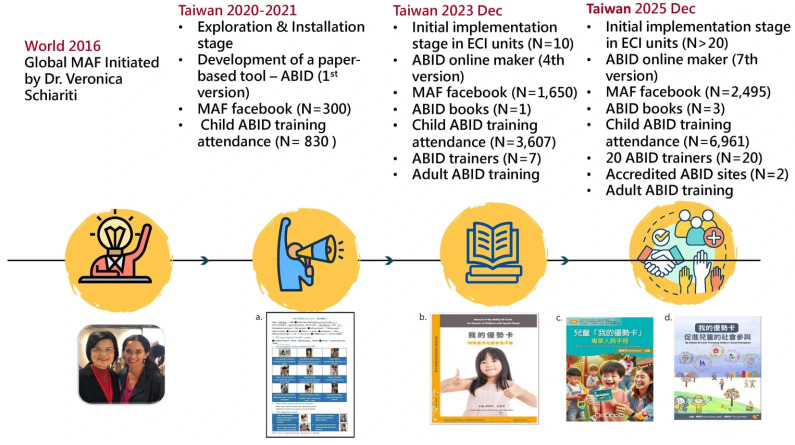
Background, development, and outcomes of My Abilities ID Card (ABID) implementation for children in Taiwan. a. ABID example. b. Manual of My Ability ID Cards for Parents of Children with Special Needs. 2022. c. My Abilities ID Cards for Children: A Professional’s Handbook. 2024. d. My Abilities ID Cards book. 2025.

Interdisciplinary MAF teams in Taiwan utilized **implementation science** [9,18,19,20] to guide the process through four stages (see Figure 3):

**(1). Exploration Stage (2020):** The team identified the target population (preschoolers with DDs) and engaged in bidirectional advocacy with the Ministry of Health and Welfare (MOHW) and education and child-care-related organizations, linking the ABID to the CRC [9]. In addition, the MAF team included a representative and diverse group of academics, clinicians, educators, administrators and international experts working in early childhood education, implementation science, and ICF-based and human rights-based approaches.

**(2). Installation Stage:** The team developed infrastructure with the support of official and private sectors, including:

**Tools:** Digital and paper blank forms of the ABID (available in Chinese and English), four checklists to enhance quality of the ABID (Checklist of the Completed My Abilities ID Cards for Children, Checklist for Producing Process of Self-report with Assist of My Abilities ID Card for Children, Checklist of the Completed My Abilities ID Cards for Adults, and Checklist for Producing Process of Self-report with Assist of My Abilities ID Card for Adults). These materials are provided on social media and are free of charge.

**Knowledge Mobilization:** A MAF Facebook group (growing from 300 to 2495 members by 2025), Line, websites, radio, newspapers, a free parent eBook (https://icf.org.tw/upload/downloads/2024010811494949A111/659c199d06921.pdf (accessed on 1 January 2023)), professional books, and publications in domestic and international conferences and journals [9].

**Workforce Development and Training:** Designing and conducting tailored training programs to fit various needs of trainees, ranging from 1 to 6 h, from introduction to practice. For qualified ABID makers, training workshops for ECI practitioners evolved from 3 h workshops (participants learn to create child ABIDs) in 2021 to 6 h ones (participants learn to create child and adult ABIDs and reflective sharing of participated parent–child dyad) in 2024. The 6 h training workshops were designed for ECI practitioners.

**(3). Initial Implementation Stage:**

**Certifying ABID Trainers and ECI Units**: Defining criteria for “Certified Training Trainers” (requiring completion of five child ABIDs and teaching videos) and “Certified ABID ECI Units” (requiring ABID integration into service procedures and supervision mechanisms), and organizing a review committee.

**Integrating ABID into Service Routines:** Completing the ABID before, during or after individualized service plans (ISPs) according to children’s needs or the ECI unit’s resources.

**(4). Scale-Up Stage:** Establishing demonstration sites, ongoing stakeholder engagement, problem-solving, and system alignment to achieve full implementation by 2028.

Additionally, in this project we applied a multifaceted mechanism of change approach to adopt ABIDs. First, by using a participatory approach from the early stages, it facilitated the understanding of the need to use ABIDs and increased the willingness to change practices. Second, each center designated ABID leaders who were respected by peers and had an influential role in changing attitudes. Third, the ABID team provided ongoing educational opportunities, bringing together experts in the field, providers, children and families, modelling the creation and implementation of ABIDs, motivating everyone to engage and contribute to the successful implementation of this new project.

## 4. Outcomes and Preliminary Results in Taiwan

This implementation study utilizes a mixed-methods approach, incorporating aggregated data from the pre-implementation phase. Conducted as part of a formal program evaluation in Taiwan, all participants engaged in the evaluation process voluntarily and anonymously. This report focuses specifically on descriptive statistics of proximal indicators—such as practitioner behaviors and communication processes—rather than distal outcomes, such as children’s participation in routine, school, or community settings. Data are presented using descriptive statistics, including percentages, medians, and ranges.

### 4.1. Training Sessions and Attendance

From April 2021 to the end of 2025, 140 workshops (online and in-person) were held, training a total attendance of 6961, including ECI professionals, elementary school teachers, trainers, college students, and parents. Even during the COVID-19 period (2021 and 2022) we held 27 online, in-person or mixed training courses with a total attendance of 1765. Due to data limitations, results are reported as total attendance counts rather than the number of unique participants.

### 4.2. Competence and Satisfaction

**Pre-Training Competence:** At the beginning of each 6 h training course in 2024 and 2025, we used Slido to collect information on participants related to their competence in the ABID. The single-choice poll question is “What best describes your experience with the My Abilities ID Card? Please select one option: (1) I have created a My Abilities ID Card; (2) I have taken a course or professional training related to the My Abilities ID Card; (3) I have read the My Abilities ID Card Parent Handbook for children or followed content from the My Abilities First Facebook group; (4) Before reading the course registration information, I had some basic understanding of the My Abilities ID Card concept; (5) Before reading the course registration information, I had no prior understanding of the My Abilities ID Card.” The 290 participants included therapists, teachers, educarers, social workers, physicians, etc. The results reveal that while 31% of practitioners had no prior understanding of the ABID, the training successfully bridged this gap (see Table 1).

**Post-Training Satisfaction**. Participant satisfaction was assessed following seven 6 h training courses (2024–2025) using an anonymous Google form. Satisfaction was measured on a 5-point scale, ranging from 5 (very satisfied) to 1 (very dissatisfied). Based on a sample of 170 respondents (after excluding missing values), over 95% reported being “very satisfied” or “satisfied” with both the training curriculum and its practical components (Table 2).

**Post-Training Self-Efficacy:** An additional single-item measure was included in the satisfaction questionnaire to assess participants’ self-efficacy. Specifically, respondents were asked: “Following this training, please rate your level of self-efficacy regarding the development of My Ability ID Cards for your clients in future practice (on a scale of 0–10, where a higher score indicates greater confidence).” This item evaluated their perceived competence in making children’s ABIDs within their clinical or educational settings. Among 168 participants, the median self-efficacy score was 8/10 (range: 3–10; mode: 8), suggesting a high level of confidence in their ability to develop ABIDs following the training.

### 4.3. Shifts in Information Sources for Assessment and Intervention Across Two Cohorts

To investigate the information sources utilized by practitioners during service delivery, anonymous pre-workshop surveys were administered via SurveyCake in 2022 and 2024. Participants were asked: “When planning or participating in the assessment and intervention of children with special needs, what are your usual sources of information?” A multi-select item addressed these primary sources, with options including: (1) personal assessments and observations; (2) information from parents/caregivers; (3) the child’s own expressions; (4) input from other teachers; (5) input from interdisciplinary team members; (6) independent online research; (7) historical records or documentation; and (8) other sources.

Analysis focused on the proportion of respondents who identified “the child’s own expressions” (Option 3) as a primary source. This proportion increased from 30% among 222 ECI practitioners in 2022 [9] to 61% among 77 ECI practitioners in 2024. While both cohorts shared similar demographic profiles—predominantly female (99% in 2022; 88% in 2024) with an age range of 20–60 years—their professional compositions differed substantially. The 2022 cohort was primarily composed of preschool teachers (*n* = 141) and educarers (*n* = 52), whereas the 2024 cohort consisted mainly of therapists (*n* = 48). The extent to which these variations in professional background influence the prioritization of the child’s voice in intervention practices warrants further investigation.

### 4.4. ABID Adoption at the Child and Service Level

At present, no policy in Taiwan mandates that ECI service providers or kindergartens incorporate the ABID into their service procedures or include it in children’s individual records. Because these data are not publicly available, the research team was only able to estimate the number of children with ABIDs through informal or private channels. According to data from the ABID online creation platform and records collected by the authors, the number of children with ABIDs was fewer than 50 in 2022 and increased to 700 by the end of 2025 for children aged under 12 years. Nevertheless, this represents less than 0.55% of preschoolers with DDs in Taiwan [21], highlighting the need for broader implementation. According to the Special Education Act in Taiwan, a child is classified as having a DD if they exhibit a significant lag or abnormality compared to their peers in one or more of the following five domains: cognitive, physical (motor), language and communication, social or emotional, and activities of daily living (self-care). While ECI services primarily focus on the birth-to-six population, the Act extends special education eligibility for this category up to age twelve to facilitate educational continuity.

Regarding the service-level adoption of the ABID for children with DDs, while over ten ECI units have initiated the protocol, only two certified ABID ECI units have successfully integrated the framework into their standard service routines. These two units are geographically distinct: one is located in a metropolitan area of western Taiwan, while the other serves a suburban region in eastern Taiwan. In the context of Taiwan, the “West” is often associated with more developed, highly populated areas, while the “East” is characterized by its mountainous terrain and rural or suburban landscapes.

### 4.5. Qualitative Feedback on Training Workshops

There was one open-question item in the satisfaction questionnaire to collect informal feedback from participants, either from the 6 h training workshops for ECI practitioners or workshops for parents of children with or without special needs. The question is “*What are your opinions or suggestions regarding My Abilities ID Card training workshops for children?*” Participants and parents highlighted many contributions, such as the ABID’s practicality, its role in fostering strengths-based conversations, and its positive impact on children’s self-awareness and motivation.

### 4.6. Integrating ABID into Undergraduate Healthcare Education

A total of 80 first- and second-year students from the Departments of Physical Therapy (PT) and Occupational Therapy at a private university participated in two 50 min sessions embedded within a foundational professional course. These sessions were facilitated by a PT professor and certified ABID trainer. The primary learning objectives were: (1) to identify the core principles of the MAF in relation to strengths-based practice and (2) to enhance self-awareness through the completion of an adult ABID self-report. The curriculum comprised a 50 min theoretical introduction to MAF and the ABID framework, followed by a 50 min hands-on practice session. Instructional strategies included interactive lectures, video demonstrations, role-playing, and reflective assignments. Qualitative and informal feedback indicated that students perceived the inclusion of the ABID as a practical and innovative element of a transformative educational approach. Furthermore, students reported that developing their own adult ABID fostered greater self-awareness and increased their perceived readiness for family-centered practice. This novel application of the ABID framework in undergraduate education demonstrates significant potential to influence future clinical practice by instilling strengths-based language early in professional development and contributing to healthcare workforce capacity.

### 4.7. Further Applications

As shown in Figure 1, the ABID serves as a facilitator of environmental and personal factors that promote the identification of abilities and supports needed related to participation. The process of developing an ABID functions both as a means and as an outcome in enhancing children’s participation and decision-making. The ABID framework aligns with Shier’s Pathways to Participation model [16]. When ECI practitioners collaborate with children to elicit and document their views in the ABID, this practice supports children in reaching the third level of participation, in which their views are taken into account. Furthermore, when the completed ABID is integrated into the care plan, this corresponds to the fourth level of participation, where children are actively involved in decision-making.

Currently, the ABID is being integrated into ISPs and transition planning to foster self-expression across educational and ECI settings in Taiwan [9]. The ABID “Little Book” format facilitates strength-focused activities at home, clinics, and schools [22,23]. Collaborative goal setting is one of the principles of family-/client-centered practice and is widely regarded as the gold standard in pediatric rehabilitation. Children’s engagement in identifying goals and experiences for participation can foster motivation, feelings of being valued and heard, self-confidence, and self-determination [24]. Collaborative goal setting using the ABID and the ICF-based Collaborative Problem Solving (ICF-CPS) model further enhances participation outcomes [9]. Within this approach, children and families are actively engaged at every stage of the intervention process: identifying participation issues, seeking explanations, prioritizing intervention goals, selecting methods, implementing interventions, and evaluating the process and outcomes [17].

As described previously, to ensure high-quality implementation, two fidelity checklists were developed. For instance, the “Checklist for Completed My Abilities ID Cards for Children” comprises 13 items, such as: “Within the Strengths I Express about Myself component, are 6–9 specific strengths clearly identified?”; “In the Supports I Need component, does each heading describe a specific support strategy or accommodation rather than a deficit?”; and “Is the ABID content sufficiently concise to allow for a comprehensive review within three minutes?” Items are rated as + (Yes), ± (Uncertain), − (No), or NA (Not Applicable). To ensure clinical fidelity, we recommend a threshold of at least 80% adherence (items scored as “Yes”) to signify that the ABID meets established quality standards. The two certified ABID ECI units currently utilize these checklists as supervision tools to maintain ongoing implementation fidelity.

## 5. Global Application of My Abilities First

We encourage every child service provider to adopt MAF in their regions. The following are some options:

### 5.1. Creating ABIDs

Practitioners are encouraged to empower children, youth, and adults with disabilities or chronic health conditions to document their unique abilities through multimodal expressions. These may include short videos (2–3 min), narrative descriptions, photographs, or illustrations, allowing for a comprehensive, person-centered representation of their functional assets. For children under the age of six, the following developmentally appropriate prompts are utilized within the ABID framework: in the component The strengths I express about myself: “What are your favorite things to do, and what are the things you like the most?”, “What are you able to do every day?”, “Can you give some examples of things that you can do and enjoy every day?” and “Who supports you to achieve your desired activities or decrease the difficulties?”; in the component The supports I need, the questions could be: “Which people or things help you do what you want to do? (e.g., play with your toys or finish your work?)” and “When you feel sad, angry, or scared, which people or things help you feel better, calm down, or feel brave?”

### 5.2. Campaigns and Advocacy

We encourage colleagues to use MAF as an advocacy tool. For example, in the context of an academic meeting, professional academy initiative, and/or disability-related campaign, you can invite children and youth in their region to create MAF or ABID videos (for an example, see https://youtu.be/wkJ4eFPs6CI?si=ZLyfeBXlrv2V7e11 (accessed on 28 July 2023)) or an A4 document, as Figure 1 (the ABID online creation platform, https://www.maf4p.com (accessed on 1 January 2024)). Then, videos or digital documents can be shared on the event’s website and social networks.

Invite one or two children and youth with a chronic health condition or disability to record a MAF short video or an ABID in their own language. You can use these materials as an advocacy effort and promote others to adopt an abilities-oriented approach in your local area.

For example, in 2020, the 32nd international conference of the European Academy of Childhood Disability conducted the first MAF campaign in an academic meeting, where children and youth shared their voices, contributing to a successful campaign. As part of many knowledge translation initiatives, Dr. Schiariti also gave a lecture to introduce the initiation of the MAF project in Taiwan. These educational sessions are currently an ongoing effort globally (https://www.youtube.com/watch?v=FDSl2WJ3sFU (accessed on 30 November 2020)).

As next steps, in June 2026, the Seventh International Conference of Taiwan Society of the ICF will hold “Amplifying Strengths, Promoting Participation: My Abilities First Story Sharing Campaign” to encourage children with special needs and their family members or professionals to share experiences and stories related to the use of the ABID or other strengths-based tools or stories related to applying strengths-based approaches. The goal of the campaign is to incorporate children’s voices in academic settings and highlight contributions of children and people with disabilities to family, school, community, employment, and public life.

### 5.3. Integration into Records

Include ABIDs in health, social welfare, or school records, ideally at the beginning of the records. It is important to show ABIDs before problems, concerns and functional limitations are discussed. Ask the members of the service team to review ABIDs before seeing children or adults with a disability.

### 5.4. Knowledge Mobilization

In order to disseminate the relevance and contribution of adopting this new approach, creative ways of knowledge mobilization are needed. It is key to use all the current ways of sharing information, including all types of social media outlets. Short messages to wider audiences are required. At the center of the dissemination strategy is education. We need to educate not only professionals but society at large. Everyone should learn and understand the importance of using positive language related to people living with disabilities across sectors—health, education, and social services.

Students, trainees, and professionals should receive information about MAF from the beginning of their entry-level college/university training. As a result, we expect that a new generation of professionals with a totally different perspective on disability—a more respectful and empathic approach—will successfully serve their communities soon.

### 5.5. Evaluating Impact

MAF encourages the adoption of an abilities-oriented approach in healthcare, social welfare, and education encounters. As with every new approach, the teams applying MAF need to check individuals’, families’, and professionals’ satisfaction and assess the impact of using this new approach. Changes in attitudes using ABIDs before and after applying this novel approach should be recorded.

## 6. Discussion

### 6.1. Contribution to Pediatric Practice

This report demonstrates how the ABID framework operationalizes children’s rights to expression and participation across ECI and educational systems. By shifting the focus from deficit-oriented assessments toward child-led strengths, the ABID aligns with contemporary pediatric principles that frame participation as both a primary outcome and a core intervention mechanism.

The Taiwanese experience confirms that the ABID can be feasibly integrated into routine service processes—such as ISPs and transition planning—acting as a complementary tool that foregrounds the child’s perspective. The preliminary evidence indicates high practitioner satisfaction and enhanced self-efficacy in facilitating strengths-based dialogue. Notably, the increased prioritization of children’s opinions suggests a vital shift toward a participatory clinical culture. Beyond its procedural utility, the ABID may serve as a relational catalyst that fosters empathy and mutual understanding among parents, children, and professionals, contributing to a fundamental attitudinal transformation in pediatric care.

### 6.2. Contribution to Healthcare Professional Education

Integrating MAF and the ABID into healthcare education promotes self-awareness, reflective learning, and strengths-based practice among students, aligning with family-centered care principles. Taiwan’s experiences are consistent with previous studies, identifying MAF as a disruptive educational approach [9,12,25].

### 6.3. Alignment with ICF & SDGs

This report demonstrates that MAF can be operationalized within a national ECI system to advance children’s rights. Unlike traditional deficit-oriented approaches, MAF aligns with the ICF framework by positioning the ABID as a facilitator of participation. The initiative also supports SDG 4 (Education) and SDG 10 (Reduced Inequalities) by empowering children to be heard.

### 6.4. Global Transferability

A key strength of the Taiwan model is the use of implementation science, which allowed systematic scaling from exploration to initial implementation. Key facilitators include advocacy, flexible ABID formats, active involvement of children and families, and sustained professional training. The increase in practitioners considering children’s opinions (from 30% to 61%) might suggest a meaningful cultural shift in clinical practice.

### 6.5. Strengths and Limitations

Strengths of this report include its comprehensive, real-world perspective, integration of quantitative and qualitative data, and transparent implementation framework. However, several limitations must be acknowledged. These include the absence of controlled comparative data and a reliance on self-reported outcomes, which may be subject to social desirability or recall bias. Furthermore, the current analysis lacks objective child outcome measures and longitudinal follow-up data. Finally, the results may reflect potential implementation bias, specifically a “strong leadership effect” within the participating units, which may influence the generalizability of the findings to settings with different organizational support structures. Broader adoption and longitudinal research are needed to further evaluate the impact. As part of the next steps, in Taiwan, a three-year longitudinal study is being conducted in collaboration with two certified ABID ECI units. A minimum of 12 child–parent dyads will be recruited to evaluate the impact of integrating the ABID into ISPs. The study aims to enhance ABID implementation fidelity, facilitate child participation, and strengthen family support systems. Ultimately, this research seeks to provide empirical evidence for the effectiveness and long-term sustainability of the ABID framework within ECI service delivery.

## 7. Conclusions

This report shows the novel implementation of the MAF initiative in Taiwan, illustrating positive adoption and overall satisfaction in ECI contexts. This report includes the cultural adaptation of MAF serving as a global model for operationalizing rights-based tools in early childhood intervention services. By combining the ABID tool with implementation science strategies, professionals might effectively shift the focus from functional deficits to strengths, ensuring that children’s voices are heard and their participation is supported. Continued efforts are needed to expand adoption, evaluate long-term outcomes, and further integrate strengths-based practices into policy and service delivery. The pioneering Taiwanese experience offers practical insights for advancing inclusive, equitable, and child-centered care worldwide.

## Figures and Tables

**Table 1:** Competence of ECI practitioners for ABID before training (*n* = 290).

Year	Created ABID Before *n* (%)	Taken Training Before *n* (%)	Have Read Information Before *n* (%)	Some Basic Understanding *n* (%)	No Prior Understanding *n* (%)	Total *n* (%)
2024	6 (4.3)	7 (5.1)	25 (18.1)	52 (37.7)	48 (34.8)	138 (100)
2025	29 (19.1)	12 (7.9)	20 (13.2)	49 (32.2)	42 (27.6)	152 (100)
Total	35 (12.1)	19 (6.6)	45 (15.5)	101 (34.8)	90 (31.0)	290 (100)

Abbreviations: ABID, My Abilities ID Card; ECI, early childhood intervention.

**Table 2:** Satisfaction with the 6 h training program among early intervention professionals (*n* = 170).

Variable	Very Satisfied *n* (%)	Satisfied *n* (%)	Acceptable *n* (%)	Dissatisfied *n* (%)
Time allocation	121 (71)	43 (25)	5 (3)	1 (1)
Content meets individual needs	125 (74)	40 (24)	4 (2)	1 (1)
Helpful for future professional work	128 (76)	38 (22)	4 (2)	0 (0)
Including exercises for adults/children	127 (75)	39 (23)	2 (1)	2 (1)
Introduction to MAF/ABID	128 (75)	36 (21)	6 (4)	0 (0)
Making procedure and checklist	125 (74)	39 (23)	5 (3)	1 (1)
Making adult ABID practice	122 (72)	42 (25)	5 (3)	1 (1)
Making children ABID demonstration	127 (77)	35 (21)	3 (2)	1 (1)
Children ABID practice (*n* = 165)	134 (81)	27 (16)	3 (2)	1 (1)
Sharing and awards (*n* = 165)	122 (74)	35 (21)	5 (3)	3 (2)
Overall interaction and responses (*n* = 165)	130 (79)	31 (19)	3 (2)	1 (1)

Abbreviations: ABID, My Abilities ID Card; MAF, My Abilities First.

## Data Availability

The data presented in this study are available upon reasonable request from the first author. The data are not publicly available due to quality improvement design.
